# Psychometric properties of the adapted measles vaccine hesitancy scale in Sudan

**DOI:** 10.1371/journal.pone.0237171

**Published:** 2020-08-06

**Authors:** Majdi M. Sabahelzain, Eve Dubé, Mohamed Moukhyer, Heidi J. Larson, Bart van den Borne, Hans Bosma

**Affiliations:** 1 Department of Public Health, School of Health Sciences, Ahfad University for Women, Omdurman, Sudan; 2 Department of Health Promotion, Care and Public Health Research Institute (CAPHRI), Maastricht University, Maastricht, the Netherlands; 3 Institut National de Santé Publique du Québec (INSPQ), Québec City, Québec, Canada; 4 Education Development and Quality Unit, College of Applied Medical Sciences, Jazan University, Jazan, Kingdom of Saudi Arabia; 5 Vaccine Confidence Project, London School of Hygiene and Tropical Medicine, Keppel Street, London, United Kingdom; 6 Department of Health Metrics Sciences, University of Washington, Seattle, Washington, University of America; 7 Department of Social Medicine, Care and Public Health Research Institute (CAPHRI), Maastricht University, Maastricht, the Netherlands; South African Medical Research Council, SOUTH AFRICA

## Abstract

**Background:**

There is a need for reliable and validated tools to identify, classify, and quantify vaccine-hesitancy in low and middle-income countries, such as Sudan. We evaluated the psychometric properties of an adapted version of the measles vaccine hesitancy scale by assessing its reliability, convergent validity, and criterion validity in Sudan. The vaccine hesitancy scale (VHS) was originally developed by the WHO/SAGE Working Group of Vaccine Hesitancy.

**Methods:**

A community-based survey among parents was conducted in February 2019 in Khartoum state. We conducted exploratory and confirmatory factor analysis to examine the structure of the adapted measles VHS (aMVHS). We computed Cronbach’s alphas, correlations with other vaccine hesitancy measurements including the Parental Attitude towards Childhood Vaccination (PACV) and the Vaccine Confidence Index (VCI), and performed a Mann-Whitney U test for assessing the reliability and the convergent and criterion validity, respectively. Moreover, to examine whether the aMVHS can predict the child’s vaccination status, the area under the curve (AUC) was estimated using receiver operator characteristic (ROC) curves.

**Results:**

The questionnaire was completed by 500 parents. Most were women (87.2%) between the ages of 20 and 47 (M = 31.15, SD = 5.74). The factor analyses indicated that the aMVHS comprises of two factors (sub-scales): 'confidence' and 'complacency'. The aMVHS sub-scales correlated weakly to moderately with the PACV and VCI scales. The area under the curve was 0.499 at most (P >0.05) and the aMVHS score did hardly differ between actually vaccinated and non-vaccinated children.

**Conclusion:**

Our findings underscore that the aMVHS and its confidence and complacency sub-scales are reliable and have a moderately good convergent validity. However, the aMVHS has a limitation in predicting the concurrent child's vaccination status. More work is needed to revise and amend this aMVHS, particularly by additionally including the 'convenience' construct and by further evaluating its validity in other contexts.

## 1. Introduction

In 2019, the World Health Organization has named vaccine hesitancy as one of the top ten global health threats, as it contributes to low vaccination coverage in both high-income and low and middle-income countries (LMICs) [1–3]. Although the definition of “vaccine hesitancy” has been debated, the Strategic Advisory Group of Experts on vaccine hesitancy (SAGE) has defined vaccine hesitancy as the "delay in acceptance or refusal of vaccines despite availability of vaccination services. Vaccine hesitancy is complex and context-specific, varying across time, place and vaccines." Moreover, vaccine hesitancy is influenced by some key factors defined in a model called the “3Cs”–Complacency (perceived risks of vaccine-preventable diseases are low and no vaccines are needed), Convenience (access issues and constraints), and Confidence (level of trust in a vaccin) [4–8].

The magnitude, factors, and causes of vaccine hesitancy as well as possible interventions to address it, have been studied and evaluated widely in high-income countries [9–12] with a paucity of research in LMIC [2, 13, 14].

There is no consensus on a global and standardized metric for measuring vaccine hesitancy [2, 14–18]. Even the existing ones were criticized by some researchers as not being comprehensive enough to address multiple dimensions of vaccine hesitancy [7, 8]. For instance; the Vaccine Confidence Index™ focuses on investigating the confidence component of the “3Cs” [1]. Additionally, the Parental Attitude towards Childhood Vaccination (PACV) scale, though it assesses three concepts (i.e. vaccination attitudes, beliefs about vaccine safety and effectiveness and behavior), these three concepts were considered part of the confidence construct [8]. Additionally, PACV-15 items has been criticized for being quite lengthy which may increase the parental burden [8, 19].

This study will contribute to the recognized need for more research in LMIC, bringing an Africa focus, in particular Khartoum state in Sudan, to the growing global portfolio of tools to measure vaccine hesitancy and its multiple domains. [2, 8, 13, 14]

To develop a global metric for vaccine hesitancy, WHO/SAGE developed tools based on its '3Cs' model which includes confidence, convenience, and complacency [15]. The WHO/SAGE recommended evaluating and researching these tools in different contexts to determine if they could be used as the basis for measuring vaccine hesitancy and be adapted to low and middle-income country settings [4, 15]. As a response to this recommendation, the Vaccine Hesitancy Scale (VHS), one of these tools which has the potential to quantify and compare vaccine hesitancy between countries and over time, has been validated and evaluated in Canada, Guatemala and recently in the United Kingdom [18, 20, 21]. Findings from these three countries revealed that VHS is a valid, reliable tool to measure vaccine hesitancy. Additionally, they recommended that more adaptation and validation of VHS is needed in different contexts [18, 20, 21]. To our knowledge, none of the existing tools to measure vaccine hesitancy have been adapted and validated in Africa.

The current study is part of a larger research project using mixed methods to investigate research gaps in vaccine hesitancy generally and particularly measles vaccine hesitancy in Khartoum state in Sudan [14]. In response to the recommendation of WHO/SAGE to evaluate vaccine hesitancy tools in different contexts, besides, to address issues that were raised about VHS in Canada, Guatemala, and the UK, we adapted the VHS and evaluated it in Sudan to further identifying quantifying and understand measles vaccine hesitancy in Sudan. Accordingly, this current study aimed at evaluating the psychometric properties of the vaccine hesitancy scale that was adapted from the scale developed by WHO/SAGE by measuring its internal consistency (reliability), construct (convergent) validity and criterion (concurrent) validity.

## 2. Methods

### 2.1. Study design

For this validation study, a community-based survey was conducted in Omdurman locality in Khartoum state in February 2019. This area was selected for the survey because a socially, culturally and economically diverse group of people, coming from all-over Sudan, live in this locality and its suburbs and have been exposed to vaccination campaigns and materials. Additionally, assessing vaccine hesitancy requires that vaccination services are available, which is the case in Omdurman.

### 2.2. Participants and sample size

The study population included parents/caregivers having a child aged 24–47 months (2–3 years). Both mothers, fathers, or any adult nominated by the parents from inside the household (household member) were eligible for the interview. If there was more than one child at the same age range, the guardian had to answer about only the youngest one. If both mother and father were available, they were asked to choose one of themselves who filled in the questionnaire.

A power analysis for the association between the measles vaccine hesitancy and the measles vaccination status (outcome) showed that at least 386 participants were needed to yield an 80% power to detect an odds ratio of 1.7 at alpha level (5%), assuming the prevalence of the outcome, the measles vaccination status among the exposed group (hesitant parents) is 50% [22]. To cover for possible drop-out due to missing information on the crucial questions, a total of 500 participants (parents/caregivers) were recruited for the study. We used stratified sampling technique for the study to collect data from parents/caregivers in two different districts (Wad Nubawi and Abo Saaeed) in Omdurman, to ensure that people from various socio-cultural and economic backgrounds were included in the validation study. In each stratum (i.e. district), parents/ caregivers were selected using the consecutive sampling; every parent/caregiver meeting the criteria of inclusion was selected until the required sample size was achieved. We appointed eight well-trained female data collectors (senior students) from Ahfad University for Women to help in collecting the data.

### 2.3. Vaccine hesitancy's measurement tools

In this study, we evaluated the psychometric properties of an adapted version of the vaccine hesitancy scale VHS which originally was developed by the WHO-SAGE and which we called the adapted Measles Vaccine Hesitancy Scale 'aMVHS'. To validate the aMVHS, two other scales were used, the Parents Attitude about Childhood Vaccination (PACV) scale and the Vaccine Confidence Index ((VCI).

#### 2.3.1. Development and adaptation of the Vaccine Hesitancy Scale (aMVHS)

This scale was developed from the 10 items of the Vaccine Hesitancy Scale (VHS) that was originally developed by WHO-SAGE (see S1 Table) [15]. The VHS is measured on a five-point Likert-type rating scale ranging from ‘strongly agree’ to ‘strongly disagree. We made changes to the 10-items VHS scale informed by recommendations from two studies conducted in two high-income countries (Canada and UK) and Guatemala (low-income country) [18, 20, 21].

To adapt and improve the VHS to the Sudanese context, we made the following changes (See the summary of changes in S1 Table); Firstly, according to the two studies in Canada and the UK, one item (i.e. item no. L10 in the VHS ‘‘my child/children does or does not need vaccines for diseases that are not common anymore”, was assessed as unreliable. For the new aMVHS, we replaced this item with item number 10 " Measles is a potentially serious disease, which can cause harm to my child" to reflect on the 'complacency' dimension in the '3C' model in the definition of vaccine hesitancy. Secondly, the convenience dimension of vaccine hesitancy (i.e. vaccine services accessibility, affordability, and availability) was not included in the VHS. A study from Sudan found that issues like limited opening hours of sessions (i.e. availability of certain vaccines 1–2 days per week) and the schedule of some vaccines' doses including measles vaccine (i.e. in the 9th and 18th months of child) were considered as contributing factors to vaccine hesitancy in Sudan [14]. Accordingly, a new item to tackle the convenience's dimension was added: "I think the measles vaccine is accessible and available when my child needs it ". Thirdly, the three studies found that the scale questions of the VHS primarily address the 'vaccine confidence' dimension. Further, it was found that the VHS consists of two factors with sub-scales characterized by ‘confidence’ and ‘risks’. The 'confidence’ items were worded positively while the items of the sub-scale ‘‘risk” are worded in the opposite direction from other items (i.e. a higher value of response in the original scale for these items indicated higher vaccine hesitancy, whereas higher values of response for other items indicated lower vaccine hesitancy). Moreover, the participants who are completing the scale may mistake this directionality. Therefore, the item L9 "I am concerned about serious adverse effects of vaccines" was replaced and worded positively which was adopted from the Vaccine Confidence Index "I think measles vaccine is safe". Fourthly, the item L5 "New vaccines carry more risks than older vaccines" was also worded negatively. We excluded it from the new scale, as we were afraid that populations diversity, cultural differences, and varying degrees of education and awareness about vaccines among parents in Sudan might influence the responses to this questions (i.e. not knowing which vaccine is the new and which vaccines is/are old). Finally, moreover, in its original version, the VHS items were targeting parents' attitudes towards childhood vaccination. We adapted these ten items so that they could be asked to measure parents' attitudes towards measles vaccination.

Similarly, as reported by the previous studies [18, 20, 21], the mean of hesitancy scores were scaled from 1–5, with higher scores indicating greater hesitancy in the aMVHS.

#### 2.3.2. Parents Attitude about Childhood Vaccination (PACV) scale

The PACV contains 15 items in three domains; immunization behavior (e.g. Have you ever delayed having your child get a shot for reasons other than illness or allergy?), perceived safety and efficacy (e.g. How concerned are you that your child might have a serious side effect from a shot?) and general attitudes and trust (e.g. It is better for my child to develop immunity by getting sick than to get a shot). In its original English-version that was used in the USA, Cronbach’s alpha coefficients for the 3 sub-domain scales were 0.74, 0.84, and 0.74, respectively [23, 24]. Consistent with prior studies, and tested in many high income and middle-income countries including Malaysia and Saudi Arabia [12, 17]. Items 1 and 2 were categorized under the behavior domain; items 7–10 were grouped under the safety and efficacy domain while items 3–6 and 11–15 in the general attitude domain responses were assigned to a score of 2 for “hesitant” responses, 1 for “not sure” responses and 0 for “non-hesitant” responses. Item scores were summed to a total score ranging from 0 to 30. Parents with children who answered ''don't know" in the above-mentioned behavior items were considered missing data because this response likely reflected poor recall rather than immunization hesitancy, as suggested by other studies. The total raw score was converted to a 0–100 scale [23, 24].

#### 2.3.3. The Vaccine Confidence Index™ (VCI)

The Vaccine Confidence Index™ uses four key questions to measure (on a five-point Likert scale) the perceived importance, safety, and effectiveness of vaccines and compatibility with religious beliefs against a mix of socio-economic and demographic factors.[1]. It was developed by the Vaccine Confidence Project and has been used globally as well as a benchmark for monitoring vaccine confidence. Following the initial global study using the VCI in 2015, the European Union commissioned the Vaccine Confidence Project to run a second and third wave using the VCI™ to measure change between 2015, 2018 and 2020. The Wellcome Trust 2018 Global Monitor ran the VCI™ in 144 countries as part of a larger study on trust in science and health. Additionally, the Philippines has run four waves of the VCI™ to measure changing confidence following the announcement of a new Dengue vaccine risk, and then to measure the impact of efforts to improve the confidence levels [25, 26].

### 2.4. Data collection

Before proceeding with the reliability and validation tests, the Arabic version of the adapted VHS scale was pre-tested with 16 parents at the Ahfad Family Center (Primary health care center) to assess its understandability and clarity of questions. The reliability and validation test was carried out on the newly developed and adapted aMVHS. PACV and the aMVHS were used in the same questionnaire. Additionally, the four VCI questions were administered.

Questions about the socio-demographic characteristics of the household were asked. Additionally, the measles vaccination status of the child aged 2–3 years, was assessed by obtaining the information from the immunization card, but when the card was not available, we relied on reports from parents about the first and the second doses.

### 2.5. Data analysis

To explore the structure of the newly developed and aMVHS, exploratory factor analysis was conducted on the construction set. Factors were extracted using varimax rotation. A confirmatory factor analysis was performed on the same set in order to test model fit. The internal consistency was assessed using Cronbach's alpha for testing the reliability of the aMVHS. To assess the convergent validity, correlation (i.e. Pearson) between the aMVHS and PACV and VCI were computed. A Mann-Whitney U test was conducted to assess the association between the aMVHS and the child's vaccination status. The receiver operator characteristic (ROC) curve was performed, besides area under the curve (AUC) for the aMVHS was computed to evaluate the ability of the aMVHS for distinguishing and predicting the child's vaccination status (Criterion validity). Data analysis was done by using IBM SPSS V.24 and IBM SPSS AmosV.23.

### Ethical consideration

The study was approved by the Ahfad University for Women's Review Board (IRB) and the National Health Research Ethics Committee at the Federal Ministry of Health in Sudan. Written informed consent was obtained from each of the participants.

## 3. Results

### 3.1. Sociodemographic characteristics of the participants and their children

Five hundreds parents completed the questionnaire. As shown in Table 1, the majority of the respondents were women (87.2%) between the ages of 20 and 47 (M = 31.15, SD = 5.74), housewives (74.6%), and had a university education (46%). Most of the respondents had 2 children (45.6%), the majority were fully vaccinated with measles vaccine (87.2%).

**Table 1:** Sociodemographic characteristics of the participants and their children (N = 500).

	N	%
**Questionnaire's Respondent**		
Mother	436	87.2
Father	23	4.6
Others	41	8.2
**Educational level of the mother**		
Not educated/illiterate	14	2.8
Primary	62	12.6
Secondary	173	34.6
University	231	46.2
Postgraduate	19	3.8
**Mother's Employment**		
Housewife	373	74.6
Student	11	2.2
Worker	14	2.8
Officer	51	10.2
Professional (Engineer, Doctor, Lawyer …etc.)	34	6.8
Self-employed	16	3.2
Others	1	0.2
**Income level (Self-ranking)**		
Very high	6	1.2
High	64	12.8
Medium	395	79.0
Low	33	6.6
Very low	2	0.4
**Number of Children < 5 years**		
1	222	44.4
2	228	45.6
3 and more	50	10.0
**Sex of the youngest child**		
Male	222	44.4
Female	278	55.6
**Measles vaccination Status of the youngest child**		
Unvaccinated	14	2.8
Partially vaccinated (one dose)	45	9.0
Fully vaccinated (two doses)	436	87.2
Don't Know/ Not sure	5	1.0

### 3.2. Structure, model fit and the internal consistency of the aMVHS

The Exploratory Factor Analysis (EFA) identified two interpretable factors with Eigenvalues (i.e. greater than one) of 4.05 for the 'confidence' factor and 1.04 for the 'complacency' factor, see Table 2. These two factors explained about half (50.8%) of the total variance of the 10 items scale. We replicated the factor analyses with an oblique rotation (Promax) and we found a similar pattern of 'confidence' and 'complacency' factors.

**Table 2:** Rotated EFA factor loading pattern and CFA standardized regression weights for the aMVHS items.

No.	aMVHS items	EFA loadings (n = 500)		CFA Standardized regression weights (n = 500)	
		Factor1	Factor2	Factor1: Confidence ^a^	Factor2: Complacency ^b^
1	Measles vaccine is important for my child to have	0.740	0.293	0.705	-
2	I think the measles vaccine is safe	0.836	0.230	0.840	-
3	I think the measles vaccine is effective	0.870	0.173	0.831	-
4	All childhood vaccines offered by the government program in my community are beneficial	0.480	0.287	0.444	-
5	Having my child vaccinated with measles vaccine is important for the health of others in my community	0.351	0.565	**-**	0.602
6	Generally, I do what my doctor or health care provider recommends about measles vaccines for my child/children.	0.144	0.768	**-**	0.646
7	The information I receive about vaccines from the vaccine program is reliable and trustworthy.	0.114	0.684	**-**	0.545
8	Getting measles vaccines is a good way to protect my child from measles	0.317	0.657	**-**	0.681
9	I think the measles vaccine is accessible and available when my child needs it	0.272	0.484	**-**	0.481
10	Measles is a potentially serious disease which can cause harm to my child	0.117	0.441	**-**	0.378

^**a**^ Confidence is defined by the SAGE as ''trust in 1) the effectiveness and safety of vaccines; 2) the system that delivers them, including the reliability and competence of the health services and health professionals and 3) the motivations of the policy-makers who decide on the needed vaccines'' [4].

^b^ SAGE explained that ''Vaccine complacency exists where perceived risks of vaccine-preventable diseases are low and vaccination is not deemed a necessary preventive action. Complacency about a particular vaccine or about vaccination in general is influenced by many factors, including other life/health responsibilities that may be seen to be more important at that point in time. Immunization program success may, paradoxically, result in complacency and ultimately, hesitancy, as individuals weigh risks of vaccines against risks of diseases that are no longer common. Self-efficacy (the self-perceived or real ability of an individual to take action to vaccinate) also influences the degree to which complacency determines hesitancy" [4].

Confirmatory factor analysis was conducted for the 10-items and it showed best goodness-of-fit for the aMVHS with two factors (RMSEA = 0.06, CFI = 0.957, TLI = 0943). As a result, this aMVHS was divided into two sub-scales, confidence (four items) and complacency (six items), see Table 2. Cronbach's alpha values were 0.82, 0.78 and 0.70 for the aMVHS scale, the confidence's sub-scale, and the complacency' sub-scale, respectively. Additionally, KMO (Kaiser-Meyer-Olkin Measure of Sampling Adequacy) was 0.87 (p value<0.0001).

### 3.3. Convergent validity

The Pearson correlations between the aMVHS including its sub-scales (i.e. ‘confidence’ and ‘complacency’) and the alternative vaccine hesitancy measures of the PACV and VCI were calculated. The aMVHS total scale correlated 0.65 (*p*-value ≤ 0.01) with the VCI and 0.23 (*p*-value ≤ 0.01) with the PACV. The Confidence's Sub-scale correlated 0.69 (*p*-value ≤ 0.01) with the VCI and 0.13 (*p*-value ≤ 0.01) with the PACV, and the VHS complacency-Sub-scale correlated 0.50 (*p*-value ≤ 0.01) with the VCI and 0.27 (*p*-value ≤ 0.01) with the PACV. The Cronbach's alpha of VCI and PACV (computed for Q3-Q15) are 0.77 and 0.62, respectively.

### 3.4. Criterion validity

The criterion validity was assessed using Mann-Whitney U test to assess the association of the aMVHS-subscales with the measles vaccination status. There were no significant differences between the aMVHS and the aMVHS-subscales' scores with the measles vaccination status of the children. The Medians (Mdn) were the same for each group (the fully vaccinated and partially vaccinated/unvaccinated), as the Mdn = 1.4, 1.25 and 1.5, for the aMVHS, ''Confidence- subscale'' and ''Complacency-subscale'', respectively. For the aMVHS, *U* = 12321.5, *Z* = -527, *p* = 0.598. For the aMVHS ''confidence", *U* = 11216.0, *Z* = -1.64, *p* = 0.101. For the aMVHS "complacency" the *U* = 12765.0, *Z* = -0.095, *p* = 0.924. Additionally, the effect sizes (*r*) were very small for the aMVHS, ''Confidence- subscale'' and ''Complacency-subscale'' (0.02, 0.07 and 0.004, respectively). Additionally, we run stratified analysis (i.e. the Mann-Whitney U test) to assess the association of the aMVHS-subscales with the measles vaccination status within each strata (i.e. the two districts) separately. We found a similar pattern in the two districts, as there were no statistical differences between the aMVHS and the aMVHS-subscales' scores with the measles vaccination status of the children within each strata (for the aMVHS, *p*-value = 0.778 and 0.706 in Wad Nubawi and Abo Saaeed districts, respectively).

### 3.5. ROC analysis

**Fig 1:**
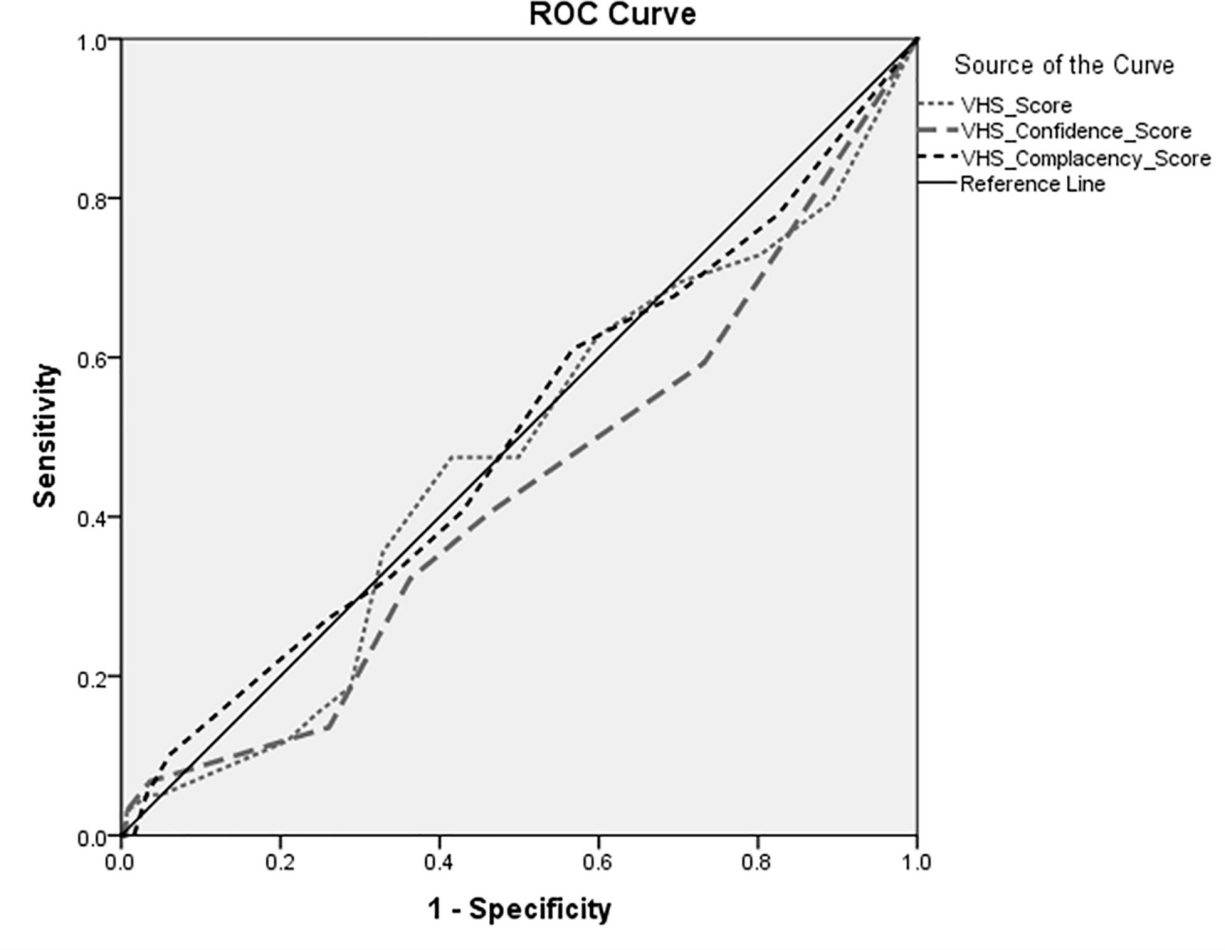
Receiver Operating Characteristic (ROC) analysis of the aMVHS and aMVHS-subscales' scores for screening of vaccine hesitancy.

The aMVHS and its subscales (confidence and complacency) were validated using ROC analysis to describe their ability to predict the child's measles vaccination status. The nonparametric analysis of the ROC for the aMVHS, confidence-subscales and complacency- subscale yielded area under the curve (AUC) of 0.474, 0.432, 0.499, respectively (P >0.05; Fig 1). This reveals that the test and its subscales cannot significantly distinguish between truly vaccinated and unvaccinated/partially vaccinated children with measles vaccine.

## 4. Discussion

In its final report on vaccine hesitancy in 2014, SAGE/WHO recommended validation of the developed survey tools on vaccine hesitancy in different contexts. However, since that time none of the existing tools to measure vaccine hesitancy have been validated in Africa. The present study aimed at evaluating the internal consistency (reliability), convergent validity, the criterion validity and the predictive value of the vaccine hesitancy scale (VHS) that was adapted from the scale developed by WHO/SAGE.

We implemented some changes to the 10- items of VHS, which were based on recommendations from previous studies [18, 20, 21].

The response rate was very high (about 100%), which resulted from using interviewer-administered questionnaires (i.e. recruiting data collectors). Our study findings showed that the adapted VHS comprises of two factors (sub-scales); 'confidence' and 'complacency'. Our findings in Sudan reflect some similarities and differences with other findings in different contexts. These findings are supported by results from three previous studies conducted to validate the VHS in Canada, UK, and Guatemala [18, 20, 21]. However, the items in each sub-scale were loaded differently than the other studies, as in our study, the 'confidence' sub-scale consisted of four items whereas the 'complacency' sub-scale consisted of six items. Apparently, these differences resulted from the adaptation of the scale. We changed the items L5 and L9 included in the sub-scale 'risk' in the original version of the VHS [18, 20, 21].

Vaccine hesitancy is complex and multi-dimensional [7, 16, 20, 24]. Unlike the other studies [18, 20, 21], our findings indicated that the adaptation that we made has improved the VHS to become bi-dimensional rather than unidimensional, as the confidence and complacency constructs are considered as part of vaccine hesitancy '3Cs' model as defined by SAGE/WHO [16]. A study in Germany has expanded on the '3Cs model' to propose a '5Cs' model and tested them locally, adding “Calculation” (i.e. individuals’ engagement in extensive information search) and “Collective responsibility” (i.e. the willingness to protect others by one's vaccination through herd immunity). However, the '5Cs' model needs more validation in differenet contexts [8]. Therefore, we suggest in the next version of the VHS to include more items on the 'convenience' dimension of the '3Cs' as described in the vaccine hesitancy's definition.

Interestingly, the construct (convergent) validity of the adapted VHS sub-scales underscored positive correlations that ranged between weak to moderate with the PACV and VCI, respectively. This can possibly be attributed to the fact that PACV and VCI focus mainly on the vaccine confidence construct [8], while the aMVHS additionally measures complacency. It is considered comparatively low, as the VHS in Canada, showed medium to large relationship when correlated with other related vaccine attitudes measures [19]. These positive correlations of the 'aMVHS' are promising as a tool to measure parental vaccine hesitancy, thus, further research is needed to evaluate the 'aMVHS' in different contexts after adding more items representing the 'convenience' dimension of vaccine hesitancy.

However, ROC analysis which yielded (AUC of 0.47), in addition to the lack of differences between the scores of the adapted MVHS and the concurrent measles vaccination status of the child, indicates the inability of the adapted VHS in predicting child’s vaccination status. Poor predictive validity of the aMVHS will not enable its use as a screening tool to guide early interventions with vaccine-hesitant parents to change their immunization behavior [21, 22].

### Limitations

The study's findings should be interpreted within the geographical, socioeconomic and sociocultural context of the study 's participants and areas. Despite this study using an Arabic version of the adapted VHS, PACV and VCI, which were forward translated by the Sudanese researchers, backward-translation was not performed. However, the pre-test showed good face validity and understandability. Findings from this study may have been limited by unintentional selection bias, as the study was conducted using a nonprobability sampling (consecutive sampling) method to recruit the participants until 500 participants were reached from both districts. Because of the time when the interviews were conducted, most of the participants were female (i.e. men were working), which led to missing fathers' perception, attitude, and role in making decisions about vaccination. In addition, about half of the female participants (46.2%) reported the completion of their university education. This figure is higher than the average of higher education attendance rates for the females (about 15% and 30% at the national and Khartoum state levels, respectively). Additionally, as noticed in the field, other household members (e.g. fathers, grandparents) were present during some of the interviews. The presence of others may have introduced a bias in participants’ responses. The study findings may have been influenced by the recall and response bias of the participants, as sometimes we rely on parental recall when there is no vaccination card to assess child’s vaccination status. As reporting perhaps biased due to social desirability. This might have even underestimated the association between vaccine hesitancy and vaccination status. However, the absence of criterion validity is not a matter of a too small N (sample size), but rather of an absent or very small difference (i.e. effect sizes) between vaccinated and non-vaccinated children. Additionally, the medians are not different for the two groups (i.e. fully vaccinated and partially/non-vaccinated. Nevertheless, given the purpose of the study to validate the adapted VHS in Sudan, we do not expect any of these limitations to have significantly impacted our results.

## Conclusion

This study aimed at evaluating the internal consistency (reliability), convergent validity, the criterion validity and the ROC curve analysis of the vaccine hesitancy scale that was adapted from developed by WHO/SAGE aMVHS. Our findings underscores that the aMVHS is reliable and that it has good convergent validity. Moreover, it constitutes two constructs of the '3Cs' model; confidence and complacency. However, the aMVHS has a limitation in predicting the concurrent child's vaccination status. More work is needed to revise and amend this aMVHS to include the 'convenience' construct as well as to evaluate its validity in other contexts.

## Supporting information

S1 Table Developing and adaptation of the Measles Vaccine Hesitancy Scale (aMVHS). (PDF)

S2 Table Frequency distribution (N = 500) of the 10 aMVHS items. (PDF)

S1 Appendix English and Arabic versions of the questionnaire. (PDF)
