# Supplementary material for: Psychometric properties of the adapted measles vaccine hesitancy scale in Sudan
Source: PLoS One. 2020 Aug 6;15(8):e0237171. doi: 10.1371/journal.pone.0237171 (PMC7410231; doi:10.1371/journal.pone.0237171)
Supplement: S1 Table — (PDF) [file pone.0237171.s001.pdf]

# S1 Table. Developing and adaptation of the Measles Vaccine Hesitancy Scale (aMVHS)

(1= Strongly agree 2= Agree 3= Don't Know/No response, 4= Disagree and 5= Strongly disagree)

| VHS | | Changes | The adapted VHS 'aMVHS' | | | | | | |
|---|---|---|---|---|---|---|---|---|---|
| No. | | | No. | | 1 | 2 | 3 | 4 | 5 |
| L1 | Childhood vaccines important for my child's health | | 1 | Measles vaccine is important for my child's health. | | | | | |
| L2 | Childhood vaccines are effective. | | 2 | I think the measles vaccine is effective. | | | | | |
| L3 | Having my child vaccinated is important for the health of others in my community | | 3 | Having my child vaccinated with the measles vaccine is important for the health of others in my community. | | | | | |

| | | | | | | | | | |
|---|---|---|---|---|---|---|---|---|---|
| L4 | All childhood vaccines offered by the government program in my community are beneficial. | | 4 | All childhood vaccines offered by the government program in my community are beneficial. | | | | | |
| L5 | New vaccines carry more risks than older vaccines | L5 was excluded from the aMVHS. However, we added item No. 5 to the 'aMVHS' in order to reflect the Convenience's dimension | 5 | I think the measles vaccine is accessible and available when my child needs it. | | | | | |
| L6 | The information I receive about vaccines from the vaccine program is reliable and trustworthy. | | 6 | The information I receive about vaccines from the vaccine program is reliable and trustworthy. | | | | | |
| L7 | Getting vaccines is a good way to protect my child from diseases. | | 7 | Getting measles vaccines is a good way to protect my child from measles. | | | | | |

| | | | | | | | | | |
|---|---|---|---|---|---|---|---|---|---|
| L8 | Generally, I do what my doctor or health care provider recommends vaccines for my child/children. | | 8 | Generally, I do what my doctor or health care provider recommends about measles vaccines for my child. | | | | | |
| L9 | I am concerned about the serious adverse effects of vaccines. | Worded positively (i.e. instead of L9) | 9 | I think the measles vaccine is safe. | | | | | |
| L10 | My child/children do or do not need vaccines for diseases that are not common anymore. | We excluded L10 from 'aMVHS' because it was found unreliable in Canada and the UK. However, we added item No. 10 in .the 'aMVHS' (i.e. instead of L10). | 10 [a] | Measles is a potentially serious disease which can cause harm to my child. | | | | | |

[a] The wording of question No.10 is negative, but the answers in the same directionality of the other 9 items of the 'aMVHS'
