# Supplementary material for: Psychometric properties of the adapted measles vaccine hesitancy scale in Sudan
Source: PLoS One. 2020 Aug 6;15(8):e0237171. doi: 10.1371/journal.pone.0237171 (PMC7410231; doi:10.1371/journal.pone.0237171)
Supplement: S2 Table — (PDF) [file pone.0237171.s002.pdf]

S2 Table. Frequency distribution (N=500) of the 10 aMVHS items

No.	aMVHS items	Strongly agree N (%)	Agree N (%)	Not sure N (%)	Disagree N (%)	Strongly disagree N (%)
1	Measles vaccine is important for my child to have	375 (75.0)	124 (24.8)	-	-	1 (0.2)
2	I think the measles vaccine is safe	290 (58.0)	197 (39.4)	10 (2)	2 (0.4)	1 (0.2)
3	I think the measles vaccine is effective	301 (60.2)	185 (37)	12 (2.4)	1 (0.2)	1 (0.2)
4	All childhood vaccines offered by the government program in my community are beneficial	187 (37.4)	284 (56.8)	18 (3.6)	10 (2)	1 (0.2)
5	Having my child vaccinated with measles vaccine is important for the health of others in my community	271 (54.2)	209 (41.8)	14 (2.8)	4 (0.8)	2 (0.4)
6	Generally, I do what my doctor or health care provider recommends about measles vaccines for my child/children.	251 (50.2)	245 (49.0)	3 (0.6)	1 (0.2)	-
7	The information I receive about vaccines from the vaccine program is reliable and trustworthy.	226 (45.2)	253 (50.6)	14 (2.8)	6 (1.2)	1 (0.2)
8	Getting measles vaccines is a good way to protect my child from measles	262 (52.4)	231 (46.2)	7 (1.4)	-	-

9	I think the measles vaccine is accessible and available when my child needs it	247 (49.4)	204 (40.8)	11 (2.2)	35 (7.0)	3 (0.6)
10	Measles is a potentially serious disease which can cause harm to my child	314 (62.8)	169 (33.8)	6 (1.2)	11 (2.2)	-
