# Supplementary material for: Psychometric properties of the adapted measles vaccine hesitancy scale in Sudan
Source: PLoS One. 2020 Aug 6;15(8):e0237171. doi: 10.1371/journal.pone.0237171 (PMC7410231; doi:10.1371/journal.pone.0237171)
Supplement: S1 Appendix — (PDF) [file pone.0237171.s003.pdf]

S3 Appendix. English and Arabic versions of the questionnaire

1/ English version

Sociodemographic data of the Household		
HH3	Respondent to Household Questionnaire	1/ Mother
		2/ Father
		3/ Others, specify……………
HH4	Total number of household members	[…….]
HH5	Number of children under age 5 years	[…….]
HH6	Head of the Household (Breadwinner)	1/ Male
		2/ Female
HH7	Age of the mother	[…….]
HH8	Age of the father	[…….]
HH9	What is the highest educational level of the mother?	1/ Not educated/illiterate
		2/ Khalwa (Religious school)
		3/ Primary
		4/ Secondary
		5/ University
		6/ Post graduate
		7/ I don't know
HH10	What is the highest educational level of the Father?	1/ Not educated/illiterate
		2/ Khalwa (Religious school)
		3/ Primary
		4/ Secondary
		5/ University
		6/ Post graduate
		7/ I don't know
HH11	What is your marital status now?	1/ Married
		2/ Widowed
		3/ Divorced
		4/ Separated
HH12	Mother's Employment	1/ Housewife
		2/ Student
		3/ Worker
		4/ Officer
		5/Professional (Engineer, Doctor, Lawyer …etc)
		6/ Self-employed
		7/ Others, Specify (………………………)
		8/ I don't know
HH13	Father's Employment	1/ Unemployed
		2/ Student
		3/ Worker

		4/ Officer
		5/Professional (Engineer, Doctor, Lawyer …etc)
		6/ Self-employed
		7/ Others, Specify (………………………)
		8/ I don't know
HH14	How do you rank your household's income level?	1/ Very high
		2/ High
		3/ Medium
		4/ Low
		5/ Very low
HH15	Age of the child of whom the respondent will answer the questionnaire (should be aged 23- 47 months (2-3 years):	[…….]
HH16	Sex of the child (who aged 23- 47 months (2-3 years)	1/ Male
		2/ Female
HH17	The rank of this child (who aged 23- 47 months (2-3 years)	[……..]
**Vaccination**		
Q1	Do you have a card where (name of the youngest child)'s vaccinations are written down?	1/ Yes, seen
		2/ Yes, not seen
		3/ No card
Q2	Measles vaccination Status of the youngest child of the respondent (should be aged 23- 35 months (2-3 years)<br><br>**(From the card or reported by the parents)**	1/ Unvaccinated **(Skip to Q3)**
		2/Partially vaccinated (One dose) **(Skip to Q4)**
		3/ Fully vaccinated (Two and more doses) **(Skip to Q5)**
		4/ I don't Know/ No response **(Skip to Q5)**
Q3	Why did you decide NOT to vaccinate your child with measles vaccine?<br>**(This question only for Parent/ Guardians who refused to vaccinate their child with measles vaccine)**	……………………………………………<br>……………………………………..................<br>……………………………………………..<br>……………………………………………<br>……………………………………………..
Q4	What are the reasons behind vaccinating your child with only one dose/ delaying the second dose of measles vaccine?<br>**(This question only for Parent/ Guardians who vaccinated their child with only one dose (Partially) or delayed the second dose of measles vaccine)**	……………………………………………<br>……………………………………..................<br>……………………………………………..<br>……………………………………………<br>……………………………………………..
Q5	In your personal opinion, why do some parents refuse to vaccinate their children with measles vaccine?	……………………………………………<br>……………………………………..................<br>……………………………………………..

		……………………………………………… ………………………………………………..
Q6	Have you ever decided not to have your child get a shot from national measles campaigns for reasons other than illness or allergy?	1/ Yes
		2/ No (Skip to Q8)
		3/ I don't know/ I don't remember
Q7	If yes, why?	……………………………………………… ……………………………………………… ……………………………………………… ……………………………………………… ………………………………………………
**Source of Information about vaccines**		
Q8	Have you ever exposed to any Pro-vaccination information/ Materials?	1/ Yes
		2/ No
Q9	From where did you get Pro-Vaccination information?<br><br>**(SELECT ALL THAT APPLY)**	1/ TV Program
		2/ Radio Program
		3/ News papers/ Magazines
		4/ Role-play/theatre
		5/ Religious lecture
		6/ Health awareness lecture
		7/ Family members
		8/ Group discussion
		9/ Home visit by heath promoter/activists
		10/ brochure/Posters
		11/ Social Media (Facebook, WhatsApp, Tweeter)
		12/ Others, specify (………………………)
Q10	Have you ever exposed to any Anti-vaccination information/ Materials?	1/ Yes
		2/ No
Q11	From where did you get Anti-Vaccination information?<br><br>**(SELECT ALL THAT APPLY)**	1/ TV Program
		2/ Radio Program
		3/ News papers/ Magazines
		4/ Role-play/theatre
		5/ Religious lecture
		6/ Health awareness lecture
		7/ Family members
		8/ Group discussion
		9/ Home visit by heath promoter/activists
		10/ brochure/Posters
		11/ Social Media (Facebook, WhatsApp, Tweeter)
		12/ Others, specify (………………………)

Vaccine Hesitancy and Vaccine Confidence Scales:

**1/ Vaccine Confidence Index**

No.		1	2	3	4	5
1	Vaccines are important for children to have	Strongly agree	Tend to agree	Don't Know/No response	Tend to disagree	Strongly disagree
2	Overall, I think vaccines are safe.	Strongly agree	Tend to agree	Don't Know/No response	Tend to disagree	Strongly disagree
3	Overall, I think vaccines are effective.	Strongly agree	Tend to agree	Don't Know/No response	Tend to disagree	Strongly disagree
4	Vaccines are compatible with my religious beliefs	Strongly agree	Tend to agree	Don't Know/No response	Tend to disagree	Strongly disagree

**2/ New Adapted Vaccine Hesitancy Scale for Measles Vaccine in Sudan**<br><br>How much do you agree with each of the following statement on vaccinations? (Scale: 1- strongly disagree, 2- disagree, 3- neither agree nor disagree, 4- agree, 5- strongly agree)						
No.		1	2	3	4	5
1	Measles vaccine is important for my child to have	Strongly dis agree	Disagree	neither agree nor disagree	Agree	Strongly agree
2	I think measles vaccine is safe	Strongly dis agree	Disagree	neither agree nor disagree	Agree	Strongly agree
3	I think measles vaccine is effective	Strongly dis agree	Disagree	neither agree nor disagree	Agree	Strongly agree
4	All childhood vaccines offered by the government program in my community are beneficial.	Strongly dis agree	Disagree	neither agree nor disagree	Agree	Strongly agree
5	Having my child vaccinated with measles vaccine is important for the health of others in my community	Strongly dis agree	Disagree	neither agree nor disagree	Agree	Strongly agree
6	Generally I do what my doctor or health care provider recommends about vaccines for my child/children.recommend about measles vaccines for my child.	Strongly dis agree	Disagree	neither agree nor disagree	Agree	Strongly agree
7	The information I receive about vaccines from the vaccine program is reliable and trustworthy.	Strongly dis agree	Disagree	neither agree nor disagree	Agree	Strongly agree
8	Getting measles vaccines is a good way to protect my child from measles	Strongly dis agree	Disagree	neither agree nor disagree	Agree	Strongly agree
9	I think measles vaccine is accessible and available when my child needs it	Strongly dis agree	Disagree	neither agree nor disagree	Agree	Strongly agree
10	Measles is a potentially serious disease which can cause harm to my child	Strongly dis agree	Disagree	neither agree nor disagree	Agree	Strongly agree

**3/ Parent Attitudes about Childhood Vaccines (PACV) Survey**												
No.												
1	Have you ever delayed having your child get a shot for reasons other than illness or allergy?	Yes		No			Don't Know					
2	Have you ever decided not to have your child get a shot for reasons other than illness or allergy?	Yes		No			Don't Know					
3	How sure are you that following the recommended shot schedule is a good idea for your child?	0	1	2	3	4	5	6	7	8	9	10
4	Children get more shots than are good for them.	Strongly agree		Agree			Not sure		Disagree		Strongly disagree	
5	believe that many of the illnesses that shots prevent are severe.	Strongly agree		Agree			Not sure		Disagree		Strongly disagree	
6	It is better for my child to develop immunity by getting sick than to get a shot.	Strongly agree		Agree			Not sure		Disagree		Strongly disagree	
7	It is better for children to get fewer vaccines at the same time.	Strongly agree		Agree			Not sure		Disagree		Strongly disagree	

8	How concerned are you that your child might have a serious side effect from a shot?	Not at all concerned	Not concerned	Not sure	Concerned	Very concerned
9	How concerned are you that any one of the childhood shots might not be safe?	Not at all concerned	Not concerned	Not sure	Concerned	Very concerned
10	How concerned are you that a shot might not prevent the disease?	Not at all concerned	Not concerned	Not sure	Concerned	Very concerned
11	If you had another infant today, would you want him/her to get all the recommended (measles) shots?	Yes	No	Don’t Know		
12	Overall, how hesitant about childhood shots would you consider yourself to be?	Not at all hesitant	Not Hesitant		Hesitant	Very hesitant
13	I trust the information I receive about shots.	Strongly agree	Agree	Not sure	Disagree	Strongly disagree
14	I am able to openly discuss my concerns about shots with my child's doctor.	Strongly agree	Agree	Not sure	Disagree	Strongly disagree
15	All things considered, how much do you trust your child’s doctor?	0 1	2 3 4	5 6	7 8	9 10

2/ Arabic version

**البيانات السكانية والأجتماعية للأسرة**		
HH1	من الذى أجاب على الأستبيان؟	1/ الأم
		2/ الأب
		3/ أخرى، حدد (..)
HH2	عدد أفراد الأسرة؟	....
HH3	عدد الأطفال دون سن الخامسة؟	....
HH4	من هو رب المنزل (عائل الأسرة)	1/ امرأة
		2/ رجل
HH5	كم عمر الأم؟	....
HH6	كم عمر الأب؟	....
HH7	ماهو مستوى تعليم الأم؟	1/ غير متعلم
		2/ خلوة
		3/ أساس
		4/ ثانوى
		5/ جامعى
		6/ ماجستير/ دكتوراة
		7/ لا أعرف
HH8	ماهو مستوى تعليم الأب؟	1/ غير متعلم
		2/ خلوة
		3/ أساس
		4/ ثانوى
		5/ جامعى
		6/ ماجستير/ دكتوراة
		7/ لا أعرف
HH9	الحالة الاجتماعية	1/ متزوجة/ متزوج
		2/أرملة
		3/مطلقة
		4/ منفصلين/ مهجورة
HH10	مهنة الأم؟	1/ ربة منزل
		2/ طالبة
		3/ عاملة
		4/ موظفة
		5/ متخصصة (مهندس، طبيب، محامى..)
		6/ عمل حر (يمتلك عمله الخاص)
		7/ أخرى (.......................................)
		8/ لا أعرف

HH11	مهنة الأب؟	1/ عاطل عن العمل
		2/ طالب
		3/ عامل
		4/ موظف
		5/ متخصص (مهندس، طبيب، محامى..)
		6/ عمل حر (يمتلك عمله الخاص)
		7/ أخرى (.......................................)
		8/ لا أعرف
HH12	**فى اعتقادك ماهو مستوى دخل اسرتك؟**	1/عالى جدا
		2/عالى
		3/ وسط
		4/ضعيف
		5/ ضعيف جدا
HH13	ماهو عمر الطفل المراد التحدث عنه (**يجب أن يكون عمره بين سنتين وثلاثة سنين أو بين 24 و47 شهر)**	....
HH14	جنس الطفل؟	1/ ولد
		2/ بنت
HH15	ترتيب الطفل بين أخوانه	....
**أسئلة عن تطعيم الحصبة**		
Q1	هل لديك كرت تطعيم لطفلك مكتوب فيه اسم الطفل (الذى عمره 2-3 سنوات)	1/ نعم (تم رؤية الكرت)
		2/ نعم (لم أرى الكرت)
		3/ لايوجد كرت
Q2	هل الطفل تم تطعيمه (تحصينه) بلقاح الحصبة (التطعيم الروتيني)<br><br>(من الكرت أو من احد الوالدين)	1/ لم يتم تطعيمه (اذهب الى السؤال رقم 3)
		2/ مطعم جزئيا (جرعة واحدة فقط) (اذهب الى السؤال رقم 4)
		3/ تطعيم كامل (جرعتين) (اذهب الى السؤال رقم 5)
Q3	لماذا قررت أن لا تطعم/ تحصن طفلك بلقاح الحصبة	....<br>..<br>..<br>..<br>..<br>..
Q4	ماهى الأسباب وراء اعطائك طفلك جرعة واحدة فقط من لقاح الحصبة؟	....<br>..<br>..<br>..<br>..<br>..
**مصادر المعلومات عن اللقاحات**		
Q5	هل سبق وأن تعرضت (قابلتك) لأي معلومات مؤيدة/ تشجع التحصين؟	1/ نعم
		2/ لا

Q6	ماهى مصادر هذه المعلومات المؤيدة للقاحات والتحصين؟	1/برنامج تلفزيوني
		2/ برنامج إذاعي
		3/ صحف/جرائد أو مجلات
		4/ عمل درامي/ مسرح
		5/ محاضرة دينية
		6/ محاضرة توعية صحية
		7/ من أفراد الأسرة
		8/ نقاش جماعي
		9/ زيارة للمنزل من قبل معززي الصحة/أو ناشطي المجتمع/
		10/ ملصقات أو منشورات
		11/ مواقع التواصل الاجتماعى (فيسبوك، واتساب، تويتر..الخ)
		12/ أخرى (حدد).................................
Q7	هل سبق وأن تعرضت (قابلتك) لأي معلومات ضد/ لا تشجع التحصين؟	1/ نعم
		2/ لا
Q8	ماهى مصادر هذه المعلومات المضادة للقاحات والتحصين؟	1/برنامج تلفزيوني
		2/ برنامج إذاعي
		3/ صحف/جرائد أو مجلات
		4/ عمل درامي/ مسرح
		5/ محاضرة دينية
		6/ محاضرة توعية صحية
		7/ من أفراد الأسرة
		8/ نقاش جماعي
		9/ زيارة للمنزل من قبل معززي الصحة/أو ناشطي المجتمع/
		10/ ملصقات أو منشورات
		11/ مواقع التواصل الاجتماعى (فيسبوك، واتساب، تويتر..الخ)
		12/ أخرى (حدد).................................

1/مؤشر الثقة فى اللقاحات

إلى أي مدى توافق على كل عبارة من العبارات التالية حول الثقة فى اللقاحات عموما!

الرقم		1	2	3	4	5
VCI1	اللقاحات مهمة للأطفال	موافق بشدة	أوافق	لا أعرف/ لا إجابة	لا أوافق	لا أوافق بشدة
VCI2	بشكل عام ، أعتقد أن اللقاحات آمنة	موافق بشدة	أوافق	لا أعرف/ لا إجابة	لا أوافق	لا أوافق بشدة
VCI3	عموما ، أعتقد أن اللقاحات فعالة	موافق بشدة	أوافق	لا أعرف/ لا إجابة	لا أوافق	لا أوافق بشدة
VCI4	تتوافق اللقاحات مع معتقداتي الدينية	موافق بشدة	أوافق	لا أعرف/ لا إجابة	لا أوافق	لا أوافق بشدة

2/ مؤشر التردد والثقة فى التطعيم بلقاح الحصبة

إلى أي مدى توافق على كل عبارة من العبارات التالية حول لقاح الحصبة؟

الرقم		1	2	3	4	5
VHS1	لقاح/تطعيم الحصبة مهم للأطفال	موافق بشدة	أوافق	لا أعرف/ لا إجابة	لا أوافق	لا أوافق بشدة
VHS 2	أعتقد أن لقاح/تطعيم الحصبة آمن	موافق بشدة	أوافق	لا أعرف/ لا إجابة	لا أوافق	لا أوافق بشدة
VHS 3	أعتقد أن لقاح/تطعيم الحصبة فعال	موافق بشدة	أوافق	لا أعرف/ لا إجابة	لا أوافق	لا أوافق بشدة
VHS 4	جميع لقاحات الطفولة التي يقدمها برنامج التحصين الحكومي في مجتمعي هي مفيدة.	موافق بشدة	أوافق	لا أعرف/ لا إجابة	لا أوافق	لا أوافق بشدة
VHS 5	إن تطعيم طفلي بلقاح الحصبة أمر مهم لصحة الآخرين في مجتمعي	موافق بشدة	أوافق	لا أعرف/ لا إجابة	لا أوافق	لا أوافق بشدة
VHS 6	عموما أفعل ما يوصي به الطبيب أو مقدم الرعاية الصحية بشأن لقاحات الحصبة لطفلي.	موافق بشدة	أوافق	لا أعرف/ لا إجابة	لا أوافق	لا أوافق بشدة
VHS 7	المعلومات التي أتلقاها عن اللقاحات من برنامج التحصين موثوقة وجديرة بالثقة	موافق بشدة	أوافق	لا أعرف/ لا إجابة	لا أوافق	لا أوافق بشدة
VHS 8	يعتبر التطعيم بلقاح الحصبة طريقة جيدة لحماية طفلي من الحصبة	موافق بشدة	أوافق	لا أعرف/ لا إجابة	لا أوافق	لا أوافق بشدة
VHS 9	أعتقد أنه من السهل الوصول للقاح الحصبة ويتوافر عندما يحتاجه طفلي	موافق بشدة	أوافق	لا أعرف/ لا إجابة	لا أوافق	لا أوافق بشدة
VHS 10	يعتبر مرض الحصبة من الأمراض الخطيرة التي يمكن أن تسبب ضرراً لطفلي	موافق بشدة	أوافق	لا أعرف/ لا إجابة	لا أوافق	لا أوافق بشدة

3/ مواقف الوالدين حول تطعيم الأطفال بلقاح الحصبة

No.												
PACV1	هل سبق لك أن تأخرت في اعطاء طفلك جرعة تطعيم الحصبة لأسباب أخرى غير المرض أو الحساسية؟	1/ نعم	2/ لا	3/ لا أعلم								
PACV2	هل سبق لك أن قررت منع طفلك من الحصول على جرعة تطعيم الحصبة لأسباب أخرى غير المرض أو الحساسية؟	1/ نعم	2/ لا	3/ لا أعلم								
PACV3	لى أي مدى أنت متأكد من أن اتباع جدول جرعات تطعيم الحصبة الموصى بها هو فكرة جيدة لطفلك؟ (جرعتين)	0	1	2	3	4	5	6	7	8	9	10
PACV4	يحصل الأطفال على جرعات أكثر من اللازم بالنسبة للقاح الحصبة	1/ موافق بشدة	2/ أوافق	3/ غير متأكد	4/ لا أوافق	5/ لا أوافق بشدة						
PACV5	أعتقد أن مرض الحصبة والذى يمكن الوقاية منه بالتطعيم هو خطير	1/ موافق بشدة	2/ أوافق	3/ غير متأكد	4/ لا أوافق	5/ لا أوافق بشدة						
PACV6	من الأفضل لطفلي أن يطور مناعته عن طريق الإصابة بالمرض بدلاً من تلقى التطعيم	1/ موافق بشدة	2/ أوافق	3/ غير متأكد	4/ لا أوافق	5/ لا أوافق بشدة						
PACV7	من الأفضل لأطفالى أن يحصلوا على عدد أقل من اللقاحات في المرة الواحدة.	1/ موافق بشدة	2/ أوافق	3/ غير متأكد	4/ لا أوافق	5/ لا أوافق بشدة						
PACV8	ما مدى تخوفك من احتمالية تعرض طفلك لأثار جانبية من لقاح الحصبة؟	1/ لست قلق/ متخوف على الاطلاق	2/ لست قلق/ متخوف	3/ غير متأكد	4/ قلق/ متخوف	5/ قلق/ متخوف جدا						

PACV9	ما مدى تخوفك من احتمالية تطعيم الحصبة أن لا يكون آمن؟ أو عدم سلامة تطعيم الحصبة؟	1/ لست قلق/ متخوف على الاطلاق	2/ لست قلق/ متخوف	3/ غير متأكد	4/ قلق/ متخوف	5/ قلق/ متخوف جدا						
PACV10	ما مدى تخوفك من احتمال عدم قدرة تطعيم الحصبة على الوقاية من مرض الحصبة؟	1/ لست قلق/ متخوف على الاطلاق	2/ لست قلق/ متخوف	3/ غير متأكد	4/ قلق/ متخوف	5/ قلق/ متخوف جدا						
PACV11	إذا كان لديك طفل آخر، هل تريده أن يحصل على كافة جرعات تطعيم الحصبة الموصى بها؟	1/ نعم	2/ لا	3/ لا أعلم								
PACV12	إلى أي مدى تعتبر نفسك مترددًا بشأن لقاح الحصبة؟	1/ لست مترددا على الاطلاق	2/لست مترددا	3/ غير متأكد	4/ متردد	5/ متردد جدا						
PACV13	أثق في المعلومات التي أتلقاها بشأن تطعيم الحصبة	1/ موافق بشدة	2/ أوافق	3/ غير متأكد	4/ لا أوافق	5/ لا أوافق بشدة						
PACV14	أنا قادرة على مناقشة مخاوفى/ تساؤلى بشكل واضح بشأن الجرعات مع الطبيب الذى يتابع مع طفلي.	1/ موافق بشدة	2/ أوافق	3/ غير متأكد	4/ لا أوافق	5/ لا أوافق بشدة						
PACV15	كل الأشياء التي تم أخذها بعين الاعتبار، إلى أي مدى تثق بالطبيب الذى يتابع مع طفلك؟	0	1	2	3	4	5	6	7	8	9	10
